# The effect of next-generation sequencing technology on complex trait research

**DOI:** 10.1111/j.1365-2362.2010.02437.x

**Published:** 2011-05

**Authors:** Aaron G Day-Williams, Eleftheria Zeggini

**Affiliations:** Wellcome Trust Sanger InstituteHinxton, Cambridge, UK

**Keywords:** Complex traits, gene mapping, genetics, next-generation sequencing, study design

## Abstract

**Background:**

Advances in the understanding of complex trait genetics have always been enabled by advances in genomic technology. Next-generation sequencing (NGS) is set to revolutionize the way complex trait genetics research is carried out.

**Results:**

NGS has multiple applications in the field of human genetics, but is accompanied by substantial study design, analysis and interpretation challenges. This review discusses key aspects of study design considerations, data handling issues and required analytical developments. We also highlight early successes in mapping genetic traits using NGS.

**Conclusion:**

NGS opens the entire spectrum of genomic alterations for the genetic analysis of complex traits and there are early publications illustrating its power. Continuing development in analytical tools will allow the promise of NGS to be realized.

## Advances in complex trait genetics

The field of complex trait genetics has progressed rapidly over the last 5 years. Irreproducible candidate gene studies examining a few single nucleotide polymorphisms (SNPs) and declaring significance at relaxed thresholds riddled the literature and represented standard study design and practice less than a decade ago. Advances in high-throughput genotyping technologies revolutionized our understanding of human genome variation, enabled better-designed studies and, with dropping costs, ultimately enabled genome-wide association scans (GWAS). GWAS transformed the field of complex trait research and have been very successful in identifying robustly associated common variants. The field is now shifting towards the study of lower frequency and rare variants, which have recently been shown to influence common traits. These studies can only be empowered by technological advances (in sequencing and rare variant typing), improved bioinformatics approaches and a better understanding of human sequence variation and thus following the historical trend of advances in human genetics.

## History of sequencing technology development

Sequencing technology advances are inextricably linked with advances in chemistry, biology, engineering and computer science [1,2]. The history of DNA sequencing has been characterized by two periods of rapid development (1970–1977; 2005–2010) with 28 intervening years of gradual progress. In December of 1977, Sanger *et al.* [3] published their “dideoxy method” that used chain-terminating nucleotide analogues to cause base-specific termination of primed DNA synthesis. This eventually became the workhorse of DNA sequencing over the next 28 years and was the foundation of the sequencing of the human genome published in 2001 [4,5]. Developments between 1977 and 2005 (when the first next-generation paper was published) were centred on increasing throughput and accuracy and decreasing cost. Important advances such as the invention of PCR [6], the development of the first automated DNA sequencer [7] and of dye-terminator sequencing [8], the release of commercial automated sequencers and the introduction of capillary electrophoresis for separating DNA molecules [9,10] paved the way for sequencing to play an important role in expanding biological knowledge.

The need for high-throughput, low-cost sequencing drove the development of massively parallel technologies, also termed next-generation sequencing (NGS) technologies. The first generation of NGS technologies achieved this using pyrosequencing (e.g. 454) [11], sequencing by ligation (e.g. SOLiD) [12] and sequencing by synthesis (e.g. Illumina, Helicos) [13–15]. Costs have already dropped dramatically from ∼$500 Million for the first human genome to ∼$10 Million using capillary sequencing to ∼$30 000 per genome using NGS in 2010.

## NGS applications in complex trait research

Next-generation sequencing can be used both for *de novo* sequencing of genomes (requiring sequence assembly) and for sample re-sequencing that compares the resulting data to the reference sequence to discover variation present in the sample. NGS greatly expands the types, sizes and frequency spectra of genomic variation amenable to analysis. In addition to genomic variation, NGS is an ideal platform to investigate gene expression below the noise level of microarrays, analyse allele-specific gene expression, investigate alternative splicing, histone modifications, transcription factor binding and achieve methylome analysis at base-pair resolution. Currently, the most widely used application of NGS in complex trait genetic studies involves SNP discovery and genotyping. NGS allows the previously unattainable, systematic discovery of low frequency variants in thousands of samples and association of these variants with phenotypes of interest. NGS can also facilitate the detection and typing of structural variants along their full size and frequency spectra, although the field is still in its infancy (but will undoubtedly rapidly progress). Large-scale experiments such as the 1000 genomes (http://www.1000genomes.org/page.php) and the UK10k (http://www.uk10k.org/) projects will provide an excellent resource of sequence data and derive variant information for the scientific community. As with the advent of high-throughput genotyping a few years ago, NGS is a technological advance that requires specialized analysis tools and carries specific study design considerations that are just now starting to be addressed.

## Analytical challenges

Although NGS can open up vast opportunities for the discovery of complex trait genetic determinants, several analytical challenges need to be addressed first. We discuss five of these issues that range from study design to downstream analyses below.

### Optimizing parameters for sequencing study design

The advent of NGS has given rise to novel study design considerations, beyond those encountered by researchers conducting large-scale genotyping experiments. Theoretically, NGS can deliver whole-genome sequencing (WGS) for individual samples, but realistically a cost-to-data equilibrium has to be reached within the context of the research question. This can be balanced by considering only a fraction of the genome and/or by pooling samples. Whole-genome, whole-exome and targeted gene/region re-sequencing offer different levels of agnosticity with inversely correlated costs. WGS approaches are appropriate when there is no *a priori* biological reason to restrict the investigation to specific regions of the genome. A similar approach was followed in GWAS and conclusively showed that common variants underlying complex traits are distributed across the genome and are not preferentially situated within genes. However, WGS can be prohibitively expensive. Whole-exome sequencing offers coverage of exons and noncoding RNAs and is the gene-centric global alternative to WGS. Targeted regional re-sequencing can be useful in fine-mapping experiments, for example following identification of robust GWAS signals. Non-WGS approaches employ sequence enrichment techniques (PCR, array-based sequence capture and in-solution capture) focusing on regions of interest. Sequence capture methods enable large-scale experiments that would not be feasible with PCR (which is difficult to multiplex, optimize and normalize, but can be highly effective). Cost efficiency can also be improved by pooling DNA samples rather than sequencing samples individually. Pools can either be indexed or nonindexed and the choice depends on the goal of the experiment. Indexing pools allow the 1:1 mapping of reads to samples, whereas nonindexed pooling does not. Nonindexed pools break the relationship between reads and samples and place an enormous burden on variant calling algorithms to disentangle true variants from NGS errors. Indexing allows individual genotyping and increased frequency estimation accuracy. The advent of NGS has also added considerations to the way in which power is calculated, for example depth of coverage has to be taken into account. Sample selection is another important aspect of study design. Depending on the research question asked, NGS experiments may be most powerful when focusing on families, unrelated individuals, selected cases and controls or individuals from the extremes of a trait distribution.

### Storing and handling data

The first pragmatic requirement of NGS analysis involves an informatics infrastructure to store, access and handle these data of unprecedented scale. Raw data from NGS platforms are base incorporation fluorescence images, analysis of which produces the base calls. As an example, a single run of an Illumina GAII sequencing machine produces approximately four terabytes (Tb) of data, but archiving of these raw image files is no longer considered necessary. Sequence alignment map (SAM) and the binary equivalent (BAM) file formats are now routinely used in production pipelines [16] across platforms. Compute and storage capacity are major considerations in NGS data generation, and the field is debating migration towards cloud computing as a possible solution.

### Mapping and aligning to the reference genome

The first step in analysing NGS data is mapping and alignment (alignment for short) of the generated small reads to a reference sequence. Computational challenges associated with this task include handling the sheer number of reads, dealing with nonunique mapping and variation in base quality, and have required algorithm development to produce efficient programs for mapping NGS reads to the reference genome [17–23]. Li and Homer [24] provide a detailed comparison of alignment algorithms. Read alignment is a computationally intensive task, and a bottleneck in the analysis of NGS data. Alignment algorithms are evolving and becoming faster, which is a necessity as lane throughput continues to grow. Correct read alignment to the reference is an extremely important step, as any mapping errors are then propagated into downstream analysis. A further major determinant of alignment algorithm performance is the completeness and accuracy of the reference genome. The current human reference genome is a composite genome from a small set of individuals and contains gaps. Recent publications of individuals’ entire genomes have revealed that they contain sequence that is not found anywhere in the reference human genome [14,15,25]. Recent unpublished work has shown an improvement in overall alignment accuracy if this novel sequence is included as part of the reference (DePristo and Li, unpublished). Emerging whole-genome NGS data will allow the construction of a more complete reference genome.

### Variant calling and genotyping

**Figure 1:**
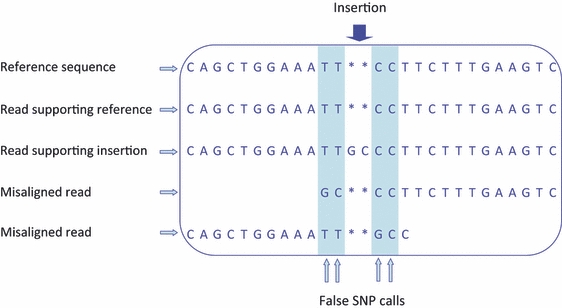
Consequences of Short Indel Misalignment on False Positive single nucleotide polymorphisms (SNP) Calling. Turquoise bars represent false positive SNPs caused by the misalignment of reads containing a two base-pair insertion relative to the reference. For the misaligned reads, since the inserted sequence occurs at the beginning/end of short reads, it is difficult for the alignment algorithms to recognize the insertion. Whereas for the correctly mapped insertion read, the inserted sequence occurs in the middle of a read with perfect matches on either side of the indel allowing the alignment algorithm correctly open a gap in the reference. Several SNPs in such a short window are hallmarks of indel misalignment.

The discovery and genotyping of sequence variants represents the cornerstone of NGS data use in complex trait genetic association studies. There are several challenges in calling true variants and distinguishing them from mapping and sequencing errors. The field has therefore been active in developing and optimizing SNP (variant) calling algorithms. We focus here on three of the sources of confounding that can substantially complicate the identification and genotyping of variants from NGS data: (i) alignment artefacts around small insertions–deletions (indels), (ii) PCR artefacts from library preparation and (iii) error profile of reads. Alignment artefacts due to indels are a major source of false positive SNP calls. The misalignment of reads containing small indels is a consequence of the fact that all reads are mapped independently and is exacerbated by algorithms that do not perform gapped alignments (M. A. Depristo et al. in preparation). The consequences of such resulting misalignment are that there will be several base mismatches with the reference around the indel. These mismatches are errors but appear to the variant calling algorithms as high-confidence SNPs because they are supported by multiple reads, all with potentially good mapping quality scores (Figure 1). Therefore, either SNP calling algorithms need to be indel-‘aware’ or potential indels should first be identified, local realignment or assembly performed around them, followed by SNP calling after completion of these initial steps. An additional challenge to true variant discovery is a consequence of errors introduced through the PCR steps in library preparation. Sequencing experiments expect that each base is equally likely to be covered and that each read represents a unique piece of DNA. However, in actuality, these null assumptions are not realized due to both variable GC content and PCR artefacts. The construction of Illumina libraries requires PCR, and during this process, a particular DNA fragment can become clonally amplified and over-represented in the library. This leads to a single piece of DNA generating a large number of nonindependent reads covering the same bases. If the PCR reaction introduces DNA replication errors, there may be numerous reads (depending on the cycle where the error was introduced) supporting the correct and the erroneous bases, thus generating what looks like a confident SNP call. The use of paired-end sequencing libraries allows researchers to identify and properly deal with these PCR-related issues. The probability that two reads from a pair-end library have the exact same outer coordinates is extremely small; therefore, reads that have *exactly* the same outer coordinates are considerably more likely to be clonal and nonindependent. Currently, the recommended way to deal with these reads is to mark them as duplicates, and only use the read in the set with the best mapping quality. In this way, if there truly is a variant at the locus, the evidence will come from independent reads. The final challenge in making good SNP calls we discuss here is the location of the variant in the reads that harbour the variant. Bases at the ends of reads can have a substantially higher error rate than bases at the beginning and middle of reads. SNP calling algorithms do consider the base quality score, but it has been shown that quality scores coming off the machines are not well-calibrated [M. A. Depristo et al. in preparation, 26,27]. This makes recalibrating the quality scores of the aligned bases before SNP calling extremely important. Even with recalibrated base qualities, the ends of reads can lead to spurious SNP calls. The approach used to filter out spurious SNP calls due to base calling errors involves clipping the end of reads, so that only the portion of reads with high-confidence bases is used for alignment and variant calling. The road to high-confidence genotyping is paved with similar challenges. Until uncertainty is minimized or eradicated, it is important to generate variant-associated quality metrics that reflect the level of confidence both at the variant level and at the individual genotype level (M. A. Depristo et al. in preparation).

### Analysing multiple low frequency and rare variants with respect to the trait of interest

**Figure 2:**
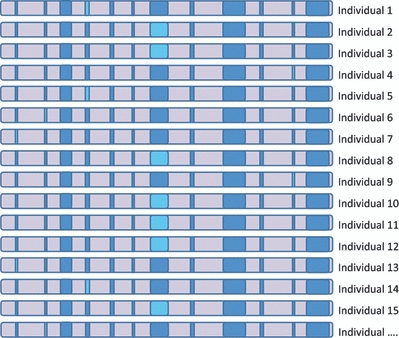
Graphical representation of allelic heterogeneity at a locus with common and low frequency/rare variant associations with a complex trait. Turquoise bars represent trait-related variants carried by different individuals at a locus of interest. Bar width represents variant frequency (wider bars denote variants with higher frequency). Single-point association analysis of the low frequency/rare variants has extremely low power, but composite analysis by considering all variants of interest locus-wide is a more powerful alternative.

The search for causal loci in rare diseases with Mendelian characteristics is conceivably relatively straightforward given full sequence data, even though analyses will need to account for sequence and calling errors that may give rise to false positive or false negative findings in the case and control samples studied. Detecting robust association with common, complex traits can be a much more complicated analytical task, as the effects sought can be very subtle. Single-point analysis of low frequency and rare variants will require hundreds of thousands of individuals in order to robustly detect modest effect sizes. To overcome this issue, powerful approaches for the aggregate analysis of multiple low frequency/rare variants across a locus that may exhibit allelic heterogeneity have recently started to emerge [28–30] (Figure 2). Analogous methods incorporating sequence-derived quality scores and genotype-specific uncertainty will also be required in order to appropriately account for possible sources of error. The interpretation of signals from such locus-wide approaches can be difficult. The paradigm of replication of association at the same variant, with the same allele, in the same direction is starting to shift in order to allow for heterogeneity across sample sets. Similarly, the combination of data in meta-analytical frameworks will have to accommodate conceivable differences in both effect size and direction of effect across strata.

## Discovery of disease genes using NGS

Research published over the last 6 months has validated the power of NGS to identify disease genes [31–35]. Different study designs and different platforms have been employed to successfully identify Mendelian disease loci, thus illustrating the robustness of data generated by NGS. Choi *et al.* reported the first use of NGS to elucidate the genetic cause of a disease through targeted re-sequencing of the exome of a single individual using NimbleGen exon-capture arrays followed by sequencing with Illumina's Genome Analyzer platform [31]. Choi *et al.* found a homozygous missense mutation in *SLC26A3* leading to a diagnosis of congenital chloride diarrhoea, although the patient was originally suspected of having Bartter syndrome. Ng *et al.* conducted a proof-of-concept experiment using Agilent exon-capture arrays and Illumina's Genome Analyzer to sequence the exomes of four unrelated individuals affected with the rare, dominantly inherited Freeman-Sheldon syndrome (FSS) and eight unaffected HapMap individuals, and unambiguously detected the previously identified gene *MYH3* responsible for FSS [32]. Ng *et al.* followed this proof-of-concept experiment with a study that identified mutations in the gene *DHODH* as the previously unidentified cause of Miller syndrome, a rare Mendelian disease. Lupski *et al.* used the SoLID NGS platform to sequence the whole genome of an individual with Charcot-Marie-Tooth neuropathy to identify the causative alleles in the gene *SH3TC2* and confirmed the causative nature of the identified mutations by directly sequencing the exons where the mutations occurred in all family members [34]. Roach *et al.* sequenced a nuclear family with two siblings affected by the recessive disorders Miller syndrome and primary ciliary dyskinesia using Complete Genomics’ service-based NGS [35] and was able to narrow down the disease-causing gene interval substantially. These five studies illustrate the power of NGS to quickly identify the causes of rare, Mendelian diseases by sequencing a small number of individuals. These are promising first steps in the use of NGS to identify disease genes, but are very different in nature and scale from the studies needed to identify susceptibility variants involved in common, complex traits.

Sequencing strategies for complex trait locus identification can differ widely and come with different sensitivity and specificity requirements (Table 1). Type 1 diabetes, a complex autoimmune disease, has provided the first example of NGS-driven disease locus identification. Nejentsev *et al.* identified four significantly associated low frequency/rare SNPs (MAF < 3%) in *IFIH1* by pooled re-sequencing of 480 cases and 480 controls using the 454 platform and thus identified *IFIH1* as the most likely causative gene in the previously associated linkage disequilibrium block that contained three other genes [36]. NGS approaches are also shedding new light into the genomic events underlying cancer. NGS of cancer genomes allows researchers to identify both SNPs and genomic rearrangements on a genome-wide scale, allowing for a greater understanding of somatic mutations. For example, NGS has provided new insight into recurrent mutations in acute myeloid leukaemia [37], acquired somatic mutations in melanoma [38], substitutions and rearrangements in lung cancer [39,40] and the evolution of substitutions and rearrangements found in breast cancer [41,42]. These early applications demonstrate the potential of NGS to help elucidate the genetics of diverse traits and diseases.

**Table 1:** Sensitivity and specificity of next-generation sequencing designs in complex disease studies

Project type	Example setting	Sequencing design	Required sensitivity	Required specificity
Sequence variant discovery	Variant identification in candidate region before genotyping follow-up	Targeted (nonindexed pools can increase cost efficiency)	High	Low
Novel association discovery	Genome-wide sequence-based association study; promising variants followed-up with genotyping	Whole-genome/Whole-exome	High	Medium
Fine-mapping established association signal	Variant identification and association testing to narrow established association signal	Targeted (indexed pools can increase cost efficiency)	Medium	High
Cancer somatic mutation	Identifying interchromosomal rearrangements, duplications, amplifications, point mutations	Whole-genome (both normal and cancer samples sequenced)	High	High

Sensitivity is defined as the ability to detect all variants present (e.g. high sensitivity ≥ high true positive rate). Specificity is defined as the correctness of called variants (e.g. high specificity ≥ high true negative rate).

## Conclusions

Next-generation sequencing has, like many technological leaps forward, realized numerous possibilities for the field of complex trait genetics. Although multiple production, study design, analysis and interpretation issues remain unresolved, progress in developing, calibrating and optimizing tools for more accurate data delivery and powerful association analysis has started to materialize. The gap in heritability left by the study of common variants can now be probed. Examples of successes are starting to emerge in the literature [36]. NGS may hold the key to linking sequence polymorphism along the full variation, frequency and effect size spectrum to polygenic phenotypes and is set to transform the way in which complex trait genetics research is carried out.

## Address

Wellcome Trust Sanger Institute, Hinxton, Cambridge, UK (A. G. Day-Williams, E. Zeggini).
